# *Thymus zygis* Essential Oil: Phytochemical Characterization, Bioactivity Evaluation and Synergistic Effect with Antibiotics against *Staphylococcus aureus*

**DOI:** 10.3390/antibiotics11020146

**Published:** 2022-01-24

**Authors:** Alexandra Coimbra, Sónia Miguel, Maximiano Ribeiro, Paula Coutinho, Lúcia Silva, Ana Paula Duarte, Susana Ferreira

**Affiliations:** 1 CICS-UBI—Health Sciences Research Centre, University of Beira Interior, Av. Infante D. Henrique, 6200-506 Covilhã, Portugal; alexandra.coimbra@hotmail.com (A.C.); spmiguel@ipg.pt (S.M.); mribeiro@ipg.pt (M.R.); coutinho@ipg.pt (P.C.); apcd@ubi.pt (A.P.D.); 2 Center of Potential and Innovation of Natural Resources, Polytechnic Institute of Guarda, 6300-559 Guarda, Portugal; 3 FibEnTech—Department of Chemistry, Fibrous Materials and Environmental Technologies, University of Beira Interior, Rua Marquês d’Ávila e Bolama, 6201-001 Covilhã, Portugal; mlas@ubi.pt

**Keywords:** *Thymus zygis*, essential oil, antioxidant activity, antimicrobial agent, *Staphylococcus aureus*, interaction with antibiotics

## Abstract

*Staphylococcus aureus* is a nosocomial bacterium causing different infectious diseases, ranging from skin and soft-tissue infections to more serious and life-threatening infections such as sepsis, meningitis and endocarditis, which may be exacerbated by antibiotic resistance. Plant products may be seen as an alternative as antibacterial agents, namely, against *S. aureus*. Thus, the aim of this work was to characterize the chemical composition and evaluate the bioactive properties of the *T. zygis* essential oil (EO), with a focus on antimicrobial activity against *S. aureus*. Gas chromatography coupled with mass spectrometry was used to assess the chemical composition of the *T. zygis* EO, and the antioxidant activity was evaluated using the DPPH method and β-carotene-bleaching assay. The antimicrobial activity against *S. aureus* strains, the interaction with different antibiotics and the attenuation of this bacterium’s virulence were evaluated. The *T. zygis* EO showed antioxidant activity acting through two different mechanisms and antibacterial activity against *S. aureus*, with antibiofilm and antihaemolytic properties. This EO also demonstrated synergistic or additive interactions in combination with ampicillin, ciprofloxacin or vancomycin against *S. aureus* strains and, in some cases, changed the antibiotic-resistance phenotype from resistant to susceptible. Therefore, the present work demonstrates the good bioactive properties of the EO of *T. zygis,* mainly the antimicrobial activity against *S. aureus*, revealing its potential to be used as an antibacterial agent.

## 1. Introduction

Antibiotics are used as the primary weapon against infections; while, at first, antibiotics were highly effective, their inappropriate use and high selective pressure have led to the emergence and spread of antibiotic-resistant bacteria [1]. In fact, antibiotic resistance has increased dramatically in recent decades and is now considered one of the greatest global health threats [2,3].

*Staphylococcus aureus* is a Gram-positive facultative anaerobic human pathogen of both nosocomial and community-acquired infections worldwide [4,5]. *S. aureus* is a commensal bacterium located on the skin and mucous membranes, but also a virulent bacterial pathogen associated with high morbidity and mortality [6,7,8,9]. This opportunistic pathogen can cause numerous acute and chronic infections [5,10], such as moderately severe skin infections, fatal pneumonia, sepsis, meningitis, endocarditis, or toxic-shock syndrome [7,11,12]. The higher rates of colonization, augmented use of surgical implants, immunosuppressive conditions, and escalation of antibiotic resistance have increased the prevalence of these infections [10]. Due to frequently occurring antibiotic resistance in *S. aureus* isolates, *S. aureus* infections are particularly problematic, and methicillin-resistant *S. aureus* (MRSA) is of utmost importance clinically. The World Health Organization states that people with MRSA infections are 64% more likely to die than people with drug-sensitive infections, and so it is on the list of microorganisms for which further investigation is critical [13].

When bacteria become resistant to first-line medicines, alternative therapies may be used [3]. The development of novel antibiotics remains a dominant approach for the treatment of bacterial-associated infections; however, this discovery is challenging [1]. Thus, it is important to explore alternative strategies and molecules to fight antibiotic-resistant *S. aureus* [1]. One possible solution is to combine antibiotics with other nonantibiotic drugs or to combine antibiotics with adjuvants or antimicrobials selected from the reservoir of bioactive compounds in nature [3,14]. 

Plant products have been used in folk medicine throughout human history and are the primary source of healthcare for much of the world’s population [2]. Hereupon, the need for novel antibacterial therapies has led to an increase in research into natural products as antibacterial agents [2]. Plants naturally produce a wide diversity of secondary metabolites, such as essential oils (EOs), that serve as defence compounds protecting against pathogens; therefore, they are important sources for the discovery of natural bioactive products [1].

Essential oils are complex blends of secondary metabolites, mainly terpenes and terpenoids [15], extracted by steam distillation, hydrodistillation or solvent extraction [16,17], which are usually stored in resin ducts, oil ducts, glands or trichomes of the plants [16]. EOs are natural products obtained from aromatic plant materials, with a broad spectrum of valuable biological properties and recognized uses in various areas (pharmaceutical, food, cosmetic and textile industries) [17,18]. They have been known to present antibacterial activity for centuries and so have been investigated for this purpose [2].

*Thymus zygis,* also known as red thyme, is predominantly found in the Mediterranean region, Asia, Southern Europe and North Africa and has been used for a long time as a spice or drug [19,20]. Its EO is known for its bioactive properties, such as antibacterial [21], antifungal [22,23] antiviral [24], antigiardial [25], insecticidal [26,27] and other properties.

Thus, considering the relevance of *S. aureus* resistance to antibiotics and the bioactive effects of *T. zygis*, this work aimed to evaluate the chemical composition of the *T. zygis* EO and to provide a better understanding of its antioxidant properties and antimicrobial activity against *S. aureus* strains, as well as cytotoxicity. The effect of the *T. zygis* EO on the virulence attenuation of *S. aureus* and also its interaction with antibiotics were evaluated.

## 2. Results

### 2.1. T. zygis EO Chemical Composition

The analysis of the chemical composition of the *T. zygis* EO through gas chromatography coupled with mass spectrometry (GC-MS) showed eighteen compounds, accounting for 94% of the total composition of the EO. The main components were identified as thymol (43.17%), carvacrol (13.00%) and *p-*cymene (10.58%) (Table 1).

### 2.2. T. zygis EO Antioxidant Activity

A very strong antioxidant activity was exhibited by the *T. zygis* EO according to the DPPH method and based on Scherer and Godoy classification [28], with IC_50_ values of 2.00 ± 0.15% (Table 2). The antioxidant activity of the *T. zygis* EO proved to have an identical effect to the Trolox standard with similar AAI values. Regarding the antioxidant activity according to a β-carotene-bleaching assay, the *T. zygis* EO showed antioxidant activity through the inhibition of lipid peroxidation (Table 2). For this reason, it can be said that the *T. zygis* EO has antioxidant activity through at least two different mechanisms, the inhibition of lipid peroxidation and sequestration of free radicals.

### 2.3. T. zygis EO Antibacterial Activity

The antimicrobial activity of the *T. zygis* EO was evaluated by using different methodologies and considering different parameters. Thus, first, it was screened through the disc-diffusion methodology, considering the EO and its volatile compounds (Table 3). According to the obtained results, higher antimicrobial activity was found against *S. aureus* ATCC 25923, with an inhibition halo of 35.10 ± 4.57 mm. The *S. aureus* SA 03/10 strain was the most resistant to the *T. zygis* EO, with an inhibition halo of 20.67 ± 1.59 mm. Concerning the evaluation of the volatile compounds of the *T. zygis* EO (Table 3), the *T. zygis* EO’s compounds released from the disc during incubation demonstrated inhibitory activity against all three *S. aureus* strains, showing inhibition halos between 16.26 ± 5.15 and 27.54 ± 4.10 mm. Regarding the study of the antimicrobial activity through the MIC determination (Table 3), the essential oil of *T. zygis* presented the same MIC value of 0.05% for the strains *S. aureus* ATCC 25922 and SA 03/10 and 0.1% against MRSA 12/08.

**Figure 1:**
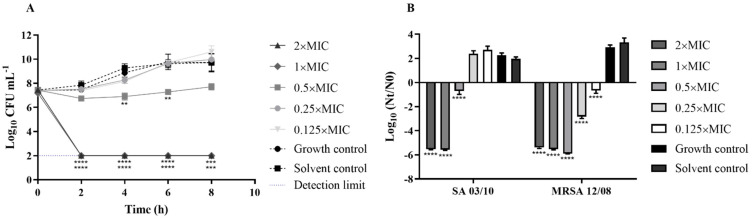
Time–kill curves for *Staphylococcus aureus* ATCC 25923 (**A**) and Log_10_ (N_t_/N_0_) of *Staphylococcus aureus* SA 03/10 and MRSA 12/08 strains at 4 h (**B**) incubated with *T. zygis* EO from 0.125× MIC to 2× MIC at 37 °C. Pointed line corresponds to the detection limit of the method. ** (*p* < 0.01); *** (*p* < 0.001); **** (*p* < 0.0001).

The antimicrobial activity can also be observed by looking at the time–kill curves showing that the *T. zygis* EO had a bactericidal effect at 1× and 2×MIC for all the strains of *S. aureus* (Figure 1). Furthermore, after 4 h of incubation, significant reductions in the logarithmic bacterial counts were observed for MRSA 12/08 with 0.5× MIC, 0.25× MIC and 0.125× MIC of *T. zygis* EO (*p* < 0.0001), while only the subinhibitory concentration of 0.5× MIC led to a significant reduction in *S. aureus* SA 03/10 (*p* < 0.0001). 

**Figure 2:**
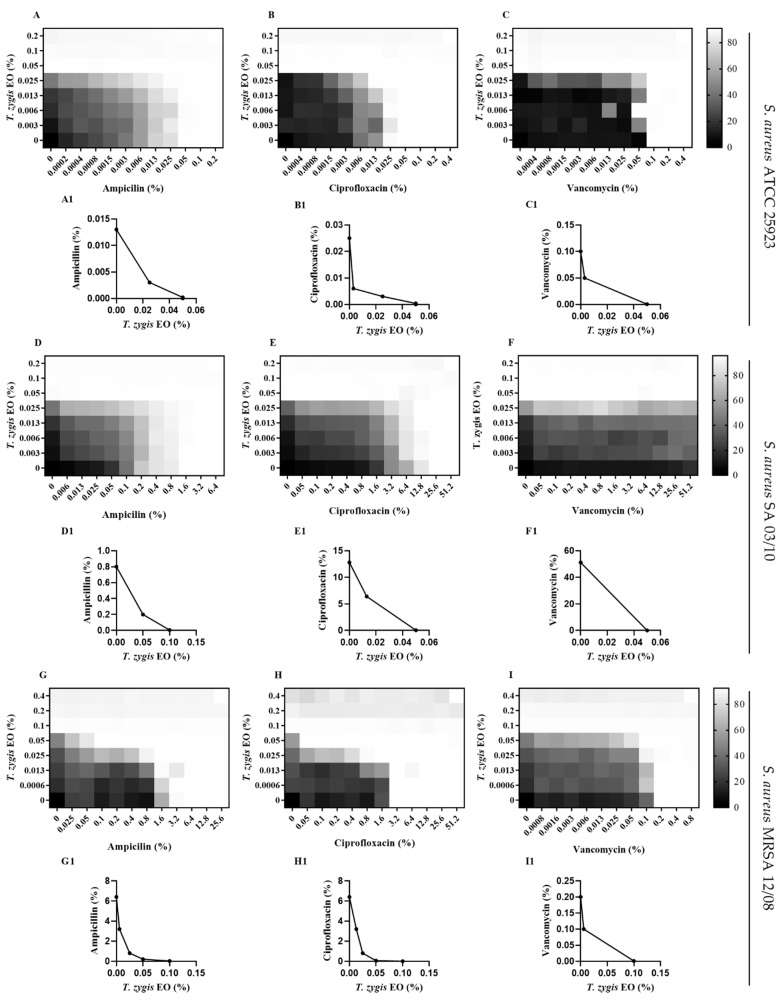
Checkerboards of *T. zygis* EO and (**A**,**D**,**G**) ampicillin (FICI = 0.74–1; 0.54–1; 0.56–1), (**B**,**E**,**H**) ciprofloxacin (FICI = 0.75–1; 0.75–1; 1) and (**C**,**F**,**I**) vancomycin (FICI = 0.31; 0.27–0.38; 0.56–1) for growth inhibition of *S. aureus* ATCC 25923, *S. aureus* SA 03/10 and *S. aureus* MRSA 12/08. The graphs (**A1**–**I1**) are the corresponding isobolograms. In the checkerboard graphics, white indicates 0% growth and black indicates 100% growth in relative terms. Points on isobolograms represent combinations of *T. zygis* EO and antibiotics (relative to their MICs alone) that exhibited > 90% growth inhibition.

The combined use of non-antibiotic compounds (known as antibiotic adjuvants) and antibiotics can be a strategy to enhance the activity of antibiotics and thus increase the susceptibility of resistant strains of bacteria [2,15]. According to the results presented in Figure 2, the *T. zygis* EO showed interactions with the antibiotics ampicillin, ciprofloxacin and vancomycin, demonstrating a synergistic (FICI ≤ 0.5) or additive (0.5 < FICI ≤ 1) effect, according to the classification of [29]. With this association, a synergic interaction occurred for the MRSA 12/08 strain with ampicillin or ciprofloxacin and the *T. zygis* EO, while the other combinations presented an additive interaction. Furthermore, the *T. zygis* EO resensitised *S. aureus* SA 03/10 to the antibiotics ampicillin, ciprofloxacin and vancomycin, and *S. aureus* MRSA 12/08, to ampicillin or ciprofloxacin. 

### 2.4. T. zygis EO Anti-Virulence Activity

**Figure 3:**
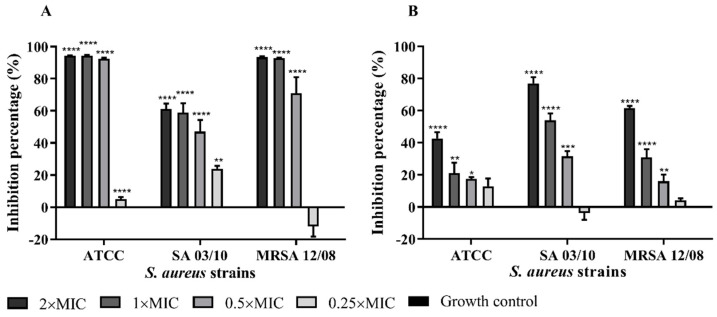
Effects of different concentrations of *T. zygis* EO on the formation of biofilms (**A**) and on elimination of pre-established biofilms (**B**) of *S. aureus* strains. Biofilm formation was estimated by the crystal-violet assay, and results are expressed as % of biofilm biomass inhibition regarding the correspondent solvent control (DMSO). * (*p* < 0.05); ** (*p* < 0.01); *** (*p* < 0.001); **** (*p* < 0.0001).

**Figure 4:**
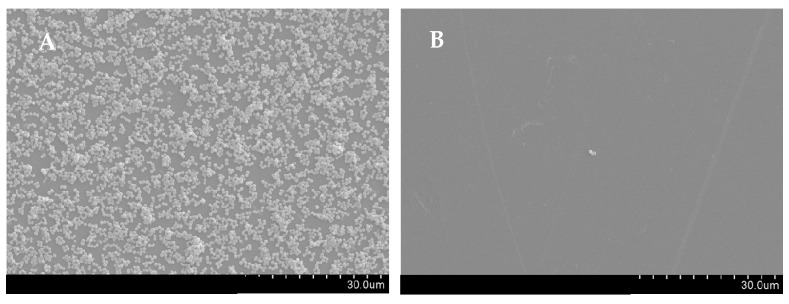
SEM micrographs showing the effect of the *T. zygis* EO on the biofilm formation: (**A**) untreated *S. aureus* MRSA 12/08; (**B**) *S. aureus* biofilm formed in the presence of *T. zygis* EO at 1× MIC. Micrographs are presented at 1500× magnification.

The essential oil of *T. zygis* was shown to affect virulence factors of *S. aureus*, such as by the inhibition of biofilm formation, the elimination of biofilms formed or affecting their haemolytic ability. The *T. zygis* EO was shown to be able to inhibit the formation of biofilms by the strains of *S. aureus* and also to partially eliminate preformed biofilms even at subinhibitory concentrations (Figure 3 and Figure 4). The effect of the essential oil of *T. zygis* in inhibiting biofilm formation was more pronounced than that in eliminating preformed biofilms, with the exception of the *S. aureus* SA 03/10 strain. 

**Figure 5:**
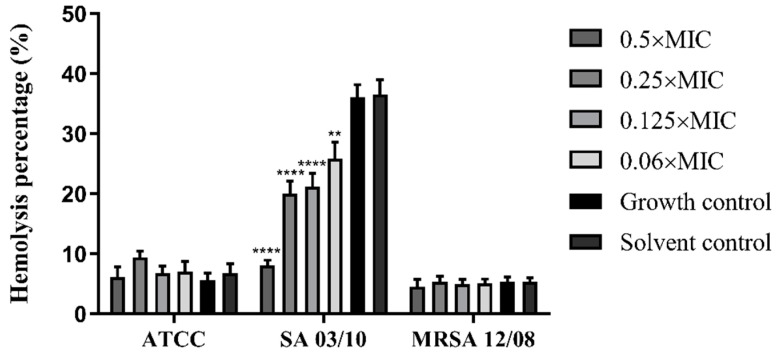
Effects of different concentrations of *T. zygis* EO on haemolytic capacity of *S. aureus* strains. ** (*p* < 0.01); **** (*p* < 0.0001).

The interference of the *T. zygis* EO with the haemolytic ability of the strains was also evaluated. Of the three strains of *S. aureus* under study, only the SA 03/10 strain demonstrated haemolytic capacity. The pre-exposure of *S. aureus* to subinhibitory *T. zygis* EO concentrations was shown to significantly reduce the haemolytic activity of *S. aureus* SA 03/10 compared to the respective controls (Figure 5) in a dose-dependent way. 

**Figure 6:**
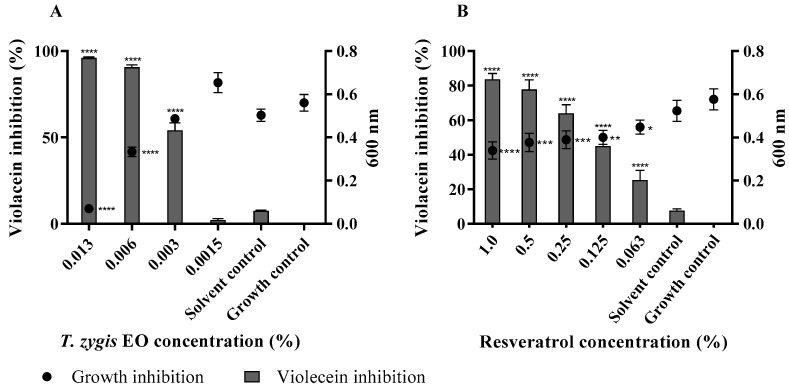
Quorum-sensing inhibition by *T. zygis* EO (**A**) and resveratrol (**B**) against *Chromobacterium violaceum*. Percentage of violacein inhibition (%) by different concentrations of EO or resveratrol and evaluation of microbial viability (OD 600 nm) after 48 h of incubation. * (*p* < 0.05); ** (*p* < 0.01); *** (*p* < 0.001); **** (*p* < 0.0001).

As quorum sensing is a mechanism that allows bacteria to control the regulation and the secretion of virulence factors, we further tested its potential inhibition by the EO [17,30]. Using *C. violaceum* as a biosensor strain to evaluate the potential of the *T. zygis* EO as a quorum-sensing inhibitor, it was observed that the concentration of 0.006% led to a significant reduction in the violet pigment production (*p* < 0.0001) without affecting the growth of *C. violaceum* (Figure 6). The concentration of 0.006% inhibited almost 100% of the violacein production, and the concentration of 0.003% inhibited approximately 50% of the pigment. It can also be observed that the *T. zygis* EO worked in a dose-dependent manner, and when the concentration of the *T. zygis* EO was decreased, the violacein inhibition also decreased. Resveratrol was used as a positive control, and there was a statistically significant inhibition of violacein production at all the concentrations under study.

**Figure 7:**
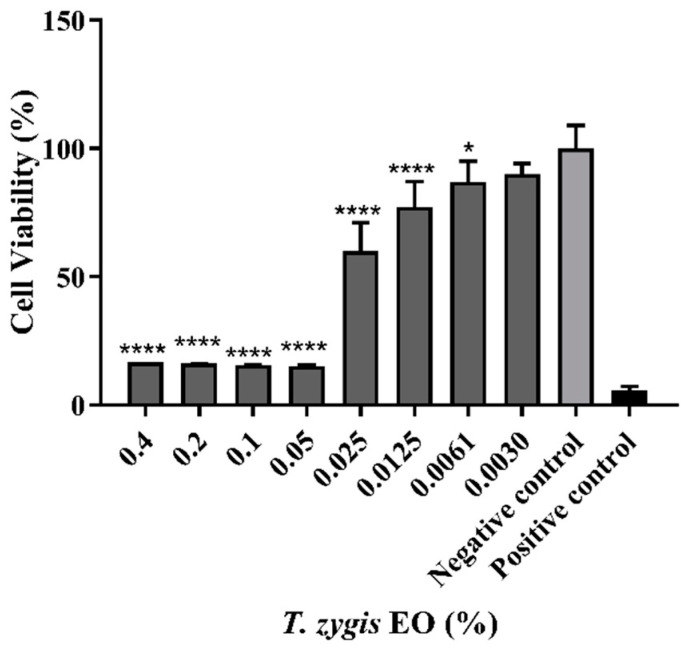
*T. zygis* EO biocompatibility for normal human dermal-fibroblast cell line measured by MTT assay after 24 h of treatment. The negative control was performed using untreated cells, and cells cultured with EtOH (96%) were used as a positive control. Results are expressed as means ± standard deviations of at least three independent experiments. * (*p* < 0.05); **** (*p* < 0.0001).

To understand the biocompatibility of the *T. zygis* EO for human cells, the effect of this essential oil was studied using a normal human dermal-fibroblast cell line for the evaluation of cytotoxicity (Figure 7). The incubation with the *T. zygis* EO reduced the viability of these cells in a dose-dependent manner. The results obtained in an MTT assay showed that the viability of NHDF was more than 70% when they were seeded in contact with the *T. zygis* EO at concentrations between 0.0125 and 0.0030%, for 24 h. Therefore, considering the ISO 10993:5–2009, it is possible to consider that such concentrations are non-cytotoxic when compared to the negative-control group (untreated cells).

## 3. Discussion

EOs have been used for centuries in perfumery, cosmetics and medicine and as part of spices and herbs in foods, and they are associated with a broad range of bioactive properties such as antibacterial and antioxidant activities [31]. The potential use of essential oils for developing promising antimicrobial agents with potential against *S. aureus* has been widely studied [32,33]. In the literature, it is described that *T. zygis* EO has antibacterial and antifungal activity against several microorganisms [34,35,36] and several other bioactive properties [15,21,37]; however, its interaction with antibiotics and several forms of antimicrobial activity required more in-depth studies. 

The bioactive properties of EOs are correlated with their compositions. Therefore, this determination is important and may allow relating the composition with the biological activities [18,38]. The composition of the *T. zygis* EO used in this work is similar to that presented in the literature, where thymol is presented as the major compound [35,36,39,40] and the cymene and carvacrol are found with considerable percentages in relation to other compounds [25,35,40,41].

Antioxidants are important because they can compete with free radicals and avoid the propagation of oxidation reactions [18]. An increase in free-radical production and decline in the activities of antioxidant-enzyme systems can damage membranes, lipids and lipoproteins and can induce DNA mutations [42,43], and the implications of lipid peroxidation can lead to a diverse number of pathological disorders [42,43,44]. The results in this work show the ability of *T. zygis* EOs to scavenge free radicals, as well as the results for the inhibition of lipid peroxidation [42], and are corroborated by other authors [19,45]. Different samples of *T. zygis* EOs were evaluated by Carrasco et al. [43], and it was shown that *T. zygis* EO with a high proportion of thymol led to better antioxidant activity, using different methodologies. As can be seen in the review of Escobar et al. [46], there are several studies showing the antioxidant activity of the isolated compound thymol. Considering that thymol is the major compound of the *T. zygis* EO under study, the high antioxidant activity may be mainly due to this compound. 

The broad spectrum of antibacterial activity of many EOs suggests a wide range of applications as antibacterial agents [2]. The *T. zygis* EO and its volatile compounds showed good antimicrobial activity against *S. aureus*, as previously described [47]. The authors related the high amount of monoterpenes in the vapour of the EO with the presented activity, as it is easier for these compounds to attack the bacterium compared to the liquid phase [47]. In accordance with our results, it was described that *T. zygis* EO also shows good inhibition of the growth of MRSA isolates [48]. The amount of thymol was also correlated with a better antimicrobial activity of *T. zygis* EO against *S. aureus*, pointing to its role in the activity of the EO [19], which may correlate with the activity observed in this work. The antimicrobial activity of the *T. zygis* EO was further validated by time–kill curves, demonstrating its bactericidal action even at subinhibitory concentrations, similarly to what has been described for other essential oils [49,50,51] and for *A. baumannii* and *K. pneumoniae* strains with *T. zygis* EO [15]. 

Drug discovery has looked to natural products for the purpose of combating infections caused by multiresistant bacteria [2]. The combination of multitarget antivirulence compounds, such as EOs, and antibiotics can help to restore the effectiveness of antibiotics, as can be seen in the review of Owen and Laird [2], and is a promising approach for combating antibiotic-resistant *S. aureus* [1,2,15]. In fact, *T. zygis* EO shows promising results in this area. The majority of the combinations of the *T. zygis* EO and antibiotics investigated mainly showed additive interactions; however, several of these additive combinations restored antibiotic sensitivity according to Clinical and Laboratory Standards Institute breakpoints [52]. The combination of the *T. zygis* EO and ampicillin, ciprofloxacin or vancomycin changed the resistance phenotype from resistant to sensitive in SA 03/10 strain (Figure 2). Regarding the two synergistic effects obtained, namely, that between the *T. zygis* EO and the antibiotic ampicillin or ciprofloxacin against the MRSA 12/08 strain, the presence of the EO also changed the resistance phenotype from resistant to sensitive in this strain. Moreover, the isobolograms show additive or synergistic effects between the combinations of *T. zygis* EO and ampicillin, ciprofloxacin or vancomycin (Figure 2). These results correlate with the synergistic interaction between thymol and ampicillin previously described [53]. In fact, as reviewed by Langeveld et al. [3], several studies showed interactions between thymol and different classes of antibiotics among different microorganisms. This interaction may be associated with the mechanism of action of thymol. Wang et al. [54] showed that thymol disrupted *S. aureus* cell membrane integrity, which may decrease cell viability and also increase the ability of other drugs to permeate the membrane [54].

The data obtained here showed that the combinations of the *T. zygis* EO and the antibiotics ampicillin, ciprofloxacin and vancomycin were able to decrease the MICs of antibiotics substantially and restore sensitivity to them, showing the EO’s potential in combating antibiotic-resistant *S. aureus* strains. As far as we know, there are no studies about the interaction of *T. zygis* EOs with antibiotics.

Antibiotic-resistant *S. aureus* poses a severe threat to human health, and antivirulence therapy is a potential antibacterial strategy for combating *S. aureus*-associated infections [1]. In fact, the *T. zygis* EO presents activity against some of the virulence factors of *S. aureus*. Among these factors, biofilms are associated with indwelling-medical-device-associated infections, endocarditis, osteomyelitis, conjunctivitis and other diseases [12]. Furthermore, biofilms can be a form of resistance to antimicrobials, host defence systems and external stresses [5,30,32]. The *T. zygis* EO decreased *S. aureus* virulence through the inhibition of biofilm formation and even the elimination of previously formed biofilms, even at subinhibitory concentrations. The antibiofilm-formation effect was further validated by SEM analysis. This antibiofilm efficacy of *T. zygis* EO was already described in the literature [40,48].

*S. aureus* is a major human pathogen that produces diverse virulence factors, such as α-haemolysin (Hla; also known as α-toxin) [4,7,9], one of the main cytotoxic agents secreted by *S. aureus*, which has been implicated in the pathogeneses of sepsis, pneumonia and severe skin infections [4,9]. The EO from *T. zygis* in the present work was shown to reduce the haemolytic capacity of *S. aureus*, comparable to other EOs that presented a similar effect [55,56]. To the best of our knowledge, this is the first report showing the efficacy of *T. zygis* EO in reducing the haemolytic capacity of *S. aureus*.

One of the antivirulence strategies aims to interfere with cell–cell communication or quorum sensing. Thus, the discovery of quorum-sensing inhibitor candidates has been presented as a step in the path toward the integration of the antivirulence strategy into the management and treatment of *S. aureus* infections [10]. The essential oil of *T. zygis* demonstrated the ability to inhibit the formation of violacein in *C. violaceum,* indicating its potential as an inhibitor of quorum sensing. Thus, the results show that, in addition to *T. zygis* having antimicrobial activity against planktonic cells, it also reduces virulence factors such as quorum sensing and biofilm formation.

The chemical composition and the biological activities of EOs are important to know, but determining their utilization limits, including their safety, is also important [18].

Similarly to our work, where a strong decrease in MTT reduction was observed for 0.25 µL/mL, the cytotoxicity of the EO of *T. zygis* against different cell lines was previously shown. In a fetal mouse-skin dendritic cell line (FSDC), no cytotoxic effect was observed at concentrations ranging from 0.08 to 0.16 µL/mL, and only for higher concentrations of the *T. zygis* EO (0.32 µL/mL) was a strong decrease in MTT reduction for the FSDC cell line noted [20]. Moreover, the EO of *T. zygis* did not cause a significant alteration in the viability of macrophages (RAW 264.7) and bovine aortic endothelial cells when compared to controls [25]. Nonetheless, when the *T. zygis* EO was tested on normal fetal lung fibroblasts (MRC-5 cell line), a significant decrease in cell viability was observed, comparable to the that for the positive control Triple antibiotic paste (metronidazole, ciprofloxacin and minocycline, at the ratio 1:1:1) [40]. In these studies, the essential oils of *T. zygis* had differences in their composition compared to the one studied in the present work, which must be considered when the biomedical application of these essential oils is envisioned.

Nonetheless, at the concentrations of *T. zygis* EO for which the cytotoxicity of the EO is low, interesting biological activities were also obtained. In the range of concentrations showing a low level of cytotoxicity, the *T. zygis* EO was shown (i) to significantly reduce the logarithmic bacterial counts of *S. aureus*, (ii) to potentiate the effects of the studied antibiotics, (iii) to inhibit the haemolytic capacity in the SA 03/10 strain or inhibit biofilm formation, and (iv) to potentiate the elimination of preformed biofilms in the *S. aureus* strains, as well as inhibiting quorum sensing in *C. violaceum*.

## 4. Materials and Methods

### 4.1. Essential Oil and Bacterial Strains

The commercial *Thymus zygis* essential oil was acquired from the company Pharmaplant (Algarve, Portugal) and was obtained by steam distillation from the aerial parts. The essential oil was protected from light and stored at 4 °C until further use.

The reference strain *S. aureus* ATCC 25923 and two clinical isolates *S. aureus* SA 03/10 and *S. aureus* MRSA 12/08 were used as test microorganisms.

### 4.2. GC-MS Analysis of the T. zygis Essential Oil

The *T. zygis* EO was analysed on an Agilent Technologies 7890A GC-System apparatus equipped with a fused silica DB-5 capillary column (Agilent J&W Column, part number: 122–5032) with a 30 m × 0.25 mm inner diameter and 0.25 µm film thickness coupled with a mass spectrometer (MS) (Agilent Technologies 5975C, Inert XL MSD) Triple-Axis detector. The operating conditions for the mass spectrometer were set as follows: ion source temperature, 250 °C; ionization voltage, 70 eV; interface temperature, 280 °C. As the carrier gas, helium was used at a flow rate of 1 mL/min. The initial oven temperature was 40 °C, with a hold time of 5 min, and it was increased to 250 °C at a rate of 5 °C/min. A 1 µL volume of *T. zygis* EO at a concentration of 10% (*v*/*v*) in dichloromethane was injected. The NIST Mass Spectral Software and Agilent GC/MSD ChemStation Software were used to calculate the relative concentration and perform identification.

### 4.3. Antioxidant Activity

The antioxidant activity of the *T. zygis* EO was evaluated using two methodologies, the 2,2-diphenyl-1-picrylhydrazyl radical (DPPH) and the β-carotene-bleaching assays, according to Coimbra et al. [57] with some adaptations. In the first one, 5 µL of each concentration of *T. zygis* EO methanolic solution was separately mixed with 195 µL of DPPH methanolic solution in 96-well microtiter plates. Methanol was used as the negative control, and Trolox (Acros Organics, Geel, Belgium) and gallic acid (Acros Organics, Geel, Belgium), as standards. The absorbances were measured at 515 nm, and the antioxidant activity was expressed through the antioxidant activity index (AAI), calculated according to:

AAI = (final concentration of DPPH in the control sample/IC_50_. (1)

In the second methodology, 56.6 μL of a *T. zygis* EO methanolic dilution was added to 943.4 μL of the emulsion. The emulsion was prepared with 500 µL of β-carotene solution at 20 mg/mL in chloroform, 40 µL of linoleic acid, 400 µL of Tween 40 and 1 mL of chloroform. Then, the chloroform was evaporated under vacuum at 45 °C, and 100 mL of oxygenated distilled water was added to form an emulsion. Butylated hydroxytoluene (BHT, purity 99%, Acros Organics, Geel, Belgium) was used as a standard, and methanol, as a negative control. The absorbances were read at 470 nm, against a blank containing an emulsion without β-carotene, and the percentage of inhibition of β-carotene oxidation was calculated using the equation:

% Inhibition = ((Abs sample^t = 1h^ − Abs control^t = 1 h^)/(Abs control^t = 0 h^ − Abs control^t = 1 h^))×100. (2)

All tests were performed in triplicate.

### 4.4. Antimicrobial Activity 

#### 4.4.1. Disc-Diffusion Method and Vapour-Phase Antimicrobial Activity Determination

The disc-diffusion method was performed to evaluate the susceptibility of the *S. aureus* strains to the *T. zygis* EO as described by Luís et al. [58]. Tetracycline at 20 µg/disc was used as a positive control. The susceptibility of the *S. aureus* strains to the volatile compounds of the *T. zygis* EO was evaluated as described by Duarte et al. [59]. These methodologies were performed with tryptic soy agar (TSA) medium. The inhibition halos were measured in millimetres, and the results are presented as means ± standard deviations. At least three independent assays were performed. 

#### 4.4.2. Determination of the Minimum Inhibitory Concentration (MIC)

The susceptibility of the *S. aureus* strains to the *T. zygis* EO was evaluated through the broth microdilution method according to Coimbra et al. [57] with modifications. Briefly, in 96-well plates, the essential oil was serially diluted with tryptic soy broth (TSB, RPD microbiology, Barcelona, Spain). Dimethyl sulfoxide (DMSO) was used as the solvent for the improvement of the solubility, with a maximum concentration of 2% (*v*/*v*) (no growth inhibition). The inoculum, with a concentration of 0.5 McFarland, was diluted in medium, and 50 µL was added to the wells to obtain a final volume of 100 µL and a concentration of 5 × 10^5^ colony-forming units (CFU)/mL per well. The MIC was determined as the lowest concentration of *T. zygis* EO without visible growth. At least three independent determinations with duplicates were performed, and the results are presented as modal values.

#### 4.4.3. Time–Kill Curves

The time–kill curve assay was performed based on Ferreira and Domingues [60] with minor modifications. Briefly, *S. aureus* strains grown overnight were used to prepare a cellular suspension to give a final cell concentration of 10^6^ CFU/mL, and it was exposed to several concentrations of *T. zygis* EO (from 0.125× to 2× MIC). A solvent control with DMSO (1% (*v*/*v*)) and growth control were also performed. The viable counts were determined by the drop-plate method at 0, 2, 4, 6 and 8 h of incubation from the tubes incubated at 37 °C. The independent experiments were performed at least thrice.

#### 4.4.4. Antibiofilm Activity of *T. zygis* EO

##### Biofilm Formation

The inhibition of biofilm formation was based on the previously described method of Stepanović et al. [61] with modifications. Briefly, *S. aureus* strains were grown overnight at 37 ˚C, at 250 rpm, in TSB. Afterwards, the turbidity of the suspension was adjusted to an OD_600 nm_ ~1.5 and diluted to achieve a final concentration in the wells of 1 × 10^7^ CFU/mL. Serial two-fold dilutions of *T. zygis* EO (0.25 to 2× MIC) were prepared in TSB, supplemented with 0.5% glucose, in 96-well flat-bottom polystyrene microtiter plates, and 100 μL of each bacterial suspension was added to the wells to obtain a final volume of 200 µL. The plates were incubated at 37 °C for 24 h. For the positive control, the bacterial suspension with medium was used, whereas for the negative control, only the culture medium was used. A solvent control in the presence of DMSO (0.125 to 1%) was also performed. After incubation, the contents of the plates were poured off, and each well was washed twice with 200 µL of distilled water to remove the loosely attached cells. The remaining attached bacteria were fixed with methanol (200 µL) for 20 min; after methanol removal, the plates were air dried. Staining was achieved with 0.1% (*w*/*v*) crystal violet (200 µL) for 10 min, the dye was removed, and the wells were washed thrice with 400 µL of distilled water. The crystal violet bound was dissolved with 33% (*v*/*v*) glacial acetic acid per well (200 µL), and the absorbance at 570 nm was determined using a microplate reader. At least five replicates of three independent experiments were conducted.

##### Biofilm Dispersion

The effect of the *T. zygis* EO on preformed biofilms was evaluated based on Duarte et al. [62] with some adaptations. Briefly, biofilms were prepared as mentioned above by inoculating 100 μL of the bacterial suspension into the wells of 96-well flat-bottom polystyrene microtiter plates containing 100 μL of TSB supplemented with 0.5% glucose. Following incubation at 37 °C for 24 h, the medium was removed and 100 μL of each *T. zygis* EO or DMSO concentration was added to the biofilm in the wells. The plates were further incubated at 37 °C for 24 h. For the positive control, 100 μL of culture medium was added, whereas for the negative control, only the culture medium was used. After incubation, the biofilm biomass was evaluated by the crystal-violet staining method as described above. At least five replicates of three independent experiments were conducted.

#### 4.4.5. Inhibition of Quorum Sensing

The anti-quorum-sensing activity of the *T. zygis* EO was assessed with the biosensor strain *Chromobacterium violaceum* ATCC 12472 and performed based on Asensio et al. [63] with some modifications. A bacterial suspension of *C. violaceum* ATCC 12472 was obtained from an overnight culture at 30 °C and 250 rpm in Luria–Bertani (LB) broth and then diluted in fresh LB broth to achieve DO_600 nm_ 0.02. *T. zygis* EO and resveratrol (positive control) were serially 2-fold diluted with LB (final concentrations of 0.0015 to 0.013% and 0.063 to 0.10%, respectively), and 500 µL of each solution was applied to 48-well flat-bottom polystyrene microtiter plates. DMSO with a final concentration of 0.125% was used as the solvent control. Then, 500 µL of the bacterial suspension was added to the wells, and the plate was incubated at 30 °C without shaking for 48 h. After incubation, 750 µL from each well was transferred to a centrifuge tube and centrifuged at 5000× *g* for 3 min. The supernatants were discarded, and the pellets were vigorously vortexed with 750 µL of DMSO to dissolve the violacein. The samples were centrifuged again at 8000× *g* for 5 min to remove the *C. violaceum* cells and to evaluate the violacein production. A 200 µL volume of violacein-containing supernatant was added into a 96-well microplate in triplicate, and the optical density at 585 nm was measured using a plate reader. The growth inhibition of *C. violaceum* was evaluated by suspending the removed cells in 750 µL of distilled water, 200 µL of the suspension was applied into a 96-well microplate in triplicate, and the absorbance was measured at 600 nm. The violacein inhibition (%) was calculated using the equation 100 − ((OD_sample_/OD_growth control_) × 100).

#### 4.4.6. Scanning Electron Microscopy (SEM)

The effect of *T. zygis* EO on biofilm formation by the strain *S. aureus* MRSA 12/08 was observed through SEM according to Luís et al. [64] with slight modifications. Biofilm formation was performed as described above but, in this case, in 24-well plates containing a polystyrene coupon with dimensions of 1 cm × 1 cm. Initially, the coupons were washed and submerged in a 70% ethanol solution overnight, followed by exposure to UV radiation for 30 min on both sides. A 500 µL volume of *T. zygis* EO (1× MIC), DMSO (0.5% (*v*/*v*), solvent control) or TSB supplemented with 0.5% glucose (growth control) was added to the plate, and 500 µL of bacterial suspension was added. After 24 h of incubation at 37 °C, the wells were washed twice with an isotonic saline solution (0.85% (*w*/*v*) NaCl) and fixed with 500 µL of 2% glutaraldehyde and 4% formaldehyde solution in PBS for 3 h at room temperature. The coupons were then carefully washed with PBS, dehydrated in a graded ethanol series (25, 50, 70, 90 and twice with 100%) and dried in a desiccator overnight. Lastly, the coupons were mounted on a stub, sputter-coated with gold and examined with a scanning electron microscope (Hitachi S-3400 N).

#### 4.4.7. Checkerboard Assay

The checkerboard method was used to test the combined effect of the *T. zygis* EO and antibiotics, according to Silva et al. [65] with some adaptations. The inoculum was prepared as described in Section 4.4.2 and the suspension was then diluted 1:67 in TSB to ensure a final cell concentration of 5 × 10^5^ CFU/mL in each well. Two microplates were prepared, one where *T. zygis* EO was successively diluted with TSB, vertically, with a final volume of 50 µL, and another plate, where successive dilutions of the antibiotics (ampicillin, ciprofloxacin or vancomycin) were carried out with TSB, in the horizontal direction. Subsequently, with a multichannel pipette, 50 µL from the plate with the antibiotic was transferred to the plate with the *T. zygis* EO, with the addition of 50 µL of inoculum to obtain a final volume of 150 µL per well. The concentrations of *T. zygis* EO and the antibiotics were selected based on the MIC values previously determined. The plate was incubated at 37 °C for 24 h. 

The results for the combined effects of the *T. zygis* EO and antibiotics were calculated and are expressed in terms of the fractional inhibitory concentration index (FICI), equal to the sum of the fractional inhibitory concentration (FIC) of the *T. zygis* EO and FICs of the antibiotics. The FIC was defined as the MIC of the EO and antibiotic in combination divided by the MIC of the EO and antibiotic used alone. If FICI ≤ 0.5, it was considered to have a synergistic effect; for 0.5 < FICI ≤ 1, there was an additive effect; 1 < FICI < 4 showed an indifferent effect; and with FICI ≥ 4, the effect was antagonistic [29].

#### 4.4.8. Effect of the *T. zygis* EO on the Haemolytic Capacity of *S. aureus*

The haemolytic activity of the *S. aureus* strains was evaluated as described by Lee et al. [5] with adaptations. Briefly, the *S. aureus* strains were grown overnight at 37 °C, at 250 rpm, for 16 h, and used to prepare a cellular suspension at a final concentration of 10^6^ CFU/mL. Tubes were prepared by adding *T. zygis* EO (0.06 to 0.5× MIC) in TSB and the cellular suspension in a final volume of 3 mL. A solvent control with DMSO (0.125% (*v*/*v*)) and a growth control were performed, and all the tubes were incubated at 37 °C for 20 h. After the incubation, 100 µL from each tube was transferred to a U-bottom 96-well plate, and 100 µL of 2% (*v*/*v*) human erythrocytes were added. A negative control (PBS without bacteria) and positive control for total haemolysis (1% (*v*/*v*) Triton-X 100) were also included. The erythrocytes were collected from one healthy volunteer into a blood collection tube with ethylenediamine tetraacetic acid (EDTA) and washed thrice with PBS, and a stock solution was prepared in the same buffer. The plate was incubated at 37 °C for 1 h and, after the incubation, was centrifuged at 1000× *g* for 5 min. A 100 µL volume of each supernatant was transferred to a 96-well flat-bottom microtiter plate, and the absorbance at 492 nm was measured. At least four replicates of three independent experiments were conducted.

### 4.5. Evaluation of T. zygis EO Biocompatibility

The cytotoxicity of the *T. zygis* EO was evaluated using normal human dermal fibroblasts (NHDF cells isolated from the dermis of adult skin and acquired from PromoCell GmbH (Heidelberg, Germany)) that were initially seeded in 96-well flat-bottom culture plates with 2×10^4^ cells/well and containing DMEM-F12 supplemented with 10% FBS. Adherent cells were grown in an incubator with a humidified atmosphere containing 5% CO_2_ at 37 °C for a day. Then, the culture medium was removed, and the cells were incubated with several concentrations of *T. zygis* EO (0.0030 and 0.4%) for 24 h. Cells cultured with EtOH (96%) were used as a positive control, and those without materials were used as a negative control. The 3-(4,5-dimethylthiazol-2-yl)-2,5-diphenyltetrazolium bromide (MTT) assay was used to monitor the cell metabolic activity. For that, the medium was removed, and a PBS solution of 5 mg/mL of MTT (50 µL in each well) was added to each sample (*n* = 5). The plate was incubated in a 5% CO_2_ atmosphere for 4 h at 37 °C. To dissolve the pigmented formazan formed, 200 µL of DMSO (0.04 N) was added to the cells for 30 min. Afterwards, a microplate reader (Biorad xMark microplate spectrophotometer, Waltham, MA, USA) was used to read the absorbance at 570 nm.

## 5. Conclusions

To summarize, this work shows that *T. zygis* EO presents good antioxidant and antimicrobial properties. *T. zygis* EO presents activity against resistant *S. aureus* strains with bactericidal activity, while showing antibiofilm and antihaemolytic activities against *S. aureus*. The *T. zygis* EO’s prospects for improving the effect of antimicrobial agents was highlighted, since the combination of the *T. zygis* EO with the antibiotics ampicillin, ciprofloxacin and vancomycin potentiated the effects of these antibiotics against the *S. aureus* strains. These results show the possible use of *T. zygis* EO as an alternative antibacterial agent for the control of *S. aureus*. 

## Figures and Tables

**Table 1:** Chemical composition of *T. zygis* essential oil according to GC-MS.

Compounds	Retention Time	Kovats Index	%
α-Thujene	10.75	929	0.73
α-Pinene	10.98	936	1.01
Camphene	11.50	950	1.19
β-Myrcene	13.14	989	1.29
α-Terpinene	13.98	1017	1.38
*p*-Cymene	14.31	1024	10.58
Limonene	14.42	1030	0.56
γ-Terpinene	15.50	1060	8.04
*Trans*-Sabinene hydrate	15.71	1098	1.14
β-Linalool	16.85	1099	3.77
Camphor	18.16	1143	1.10
*Trans*-pinocarveol	18.31	1140	0.89
Borneol	18.90	1166	3.79
4-Terpineol	19.21	1177	0.46
Thymol	22.78	**1290**	**43.17**
Carvacrol	22.99	1300	13.00
β-Caryophyllene	26.03	1420	1.43
Caryophyllene oxide	30.01	1581	0.59

The value in bold represents the major component of *T. zygis* EO.

**Table 2:** Results for antioxidant activity of *T. zygis* EO and standards measured using the DPPH method (mean ± standard deviation) and β-carotene-bleaching assay (results expressed as medians).

	DPPH Method			β-Carotene-Bleaching Assay
Samples	IC_50_ (%)	AAI	Antioxidant Activity Classification	IC_50_ (%)
*T. zygis*	2.00 ± 0.15	12.87 ± 3.65	Very strong	0.27
Gallic acid	2.14 ± 0.39	22.16 ± 3.53	Very strong	-
Trolox	3.26 ± 1.21	15.02 ± 0.64	Very strong	-
BHT	-	-	-	0.10

*T. zygis* EO IC_50_s are presented as % (*v*/*v*) and standards as % (*w*/*v*); AAI—Antioxidant activity index; BHT—Butylated hydroxytoluene.

**Table 3:** Diameters of inhibition zones for disc-diffusion method and volatile compounds of *T. zygis* EO.

	Inhibition Zone (mm)			MIC (%)	
Species	*T. zygis* (10 µL/Disc)	Tetracycline (20 µg/Disc)	Volatile Compounds (10 µL/Disc)	*T. zygis*	Tetracycline
*S. aureus* ATCC 25923	35.10 ± 4.57	31.17 ± 2.73	27.54 ± 4.10	0.05	0.013
*S. aureus* SA 03/10	20.67 ± 1.59	8.24 ± 0.49	16.26 ± 5.15	0.05	6.4
*S. aureus* MRSA 12/08	30.93 ± 4.64	8.42 ± 0.75	16.45 ± 3.63	0.1	6.4

MIC—minimum inhibitory concentration of *T. zygis* EO (%, *v*/*v*) and tetracycline (%, *w*/*v*). Values for inhibition zone are presented as means ± standard deviations, and MIC values of *T. zygis* EO and tetracycline are presented as modal values.

## Data Availability

Data are contained within the text.

## References

[1] Wu S.C., Liu F., Zhu K., Shen J.Z. (2019). Natural products that target virulence factors in antibiotic-resistant *Staphylococcus aureus*. J. Agric. Food Chem..

[2] Owen L., Laird K. (2018). Synchronous application of antibiotics and essential oils: Dual mechanisms of action as a potential solution to antibiotic resistance. Crit. Rev. Microbiol..

[3] Langeveld W.T., Veldhuizen E.J.A., Burt S.A. (2014). Synergy between essential oil components and antibiotics: A review. Crit. Rev. Microbiol..

[4] Lee K., Lee J.H., Kim S.I., Cho M.H., Lee J. (2014). Anti-biofilm, anti-hemolysis, and anti-virulence activities of black pepper, cananga, myrrh oils, and nerolidol against *Staphylococcus aureus*. Appl. Microbiol. Biotechnol..

[5] Lee J.H., Kim Y.G., Park J.G., Lee J. (2017). Supercritical fluid extracts of *Moringa oleifera* and their unsaturated fatty acid components inhibit biofilm formation by *Staphylococcus aureus*. Food Control.

[6] Zhang Y., Wang J., Dong J., Wei J., Wang Y., Dai X., Wang X., Luo M., Tan W., Deng X. (2013). Inhibition of α-toxin production by subinhibitory concentrations of naringenin controls *Staphylococcus aureus* pneumonia. Fitoterapia.

[7] Otto M. (2014). *Staphylococcus aureus* toxins. Curr. Opin. Microbiol..

[8] Vandenesch F., Lina G., Henry T. (2012). *Staphylococcus aureus* hemolysins, bi-component leukocidins, and cytolytic peptides: A redundant arsenal of membrane-damaging virulence factors?. Front. Cell. Infect. Microbiol..

[9] Singh V., Phukan U.J. (2019). Interaction of host and *Staphylococcus aureus* protease-system regulates virulence and pathogenicity. Med. Microbiol. Immunol..

[10] Quave C.L., Horswill A.R. (2014). Flipping the switch: Tools for detecting small molecule inhibitors of staphylococcal virulence. Front. Microbiol..

[11] Korem M., Gov Y., Shirron N., Shuster A., Rosenberg M. (2007). Alcohol increases hemolysis by staphylococci. FEMS Microbiol. Lett..

[12] Cheung G.Y.C., Bae J.S., Otto M. (2021). Pathogenicity and virulence of *Staphylococcus aureus*. Virulence.

[13] World Health Organization WHO’s First global Report on Antibiotic Resistance Reveals Serious, Worldwide Threat to Public Health. https://www.who.int/news/item/30-04-2014-who-s-first-global-report-on-antibiotic-resistance-reveals-serious-worldwide-threat-to-public-health..

[14] Ju J., Xie Y., Yu H., Guo Y., Cheng Y., Qian H., Yao W. (2020). Synergistic interactions of plant essential oils with antimicrobial agents: A new antimicrobial therapy. Crit. Rev. Food Sci. Nutr..

[15] Vázquez-Ucha J.C., Martínez-Guitián M., Lasarte-Monterrubio C., Conde-Pérez K., Arca-Suárez J., Álvarez-Fraga L., Pérez A., Crecente-Campo J., Alonso M.J., Bou G. (2020). *Syzygium aromaticum* (clove) and *Thymus zygis* (thyme) essential oils increase susceptibility to colistin in the nosocomial pathogens *Acinetobacter baumannii* and *Klebsiella pneumoniae*. Biomed. Pharmacother..

[16] Raut J.S., Karuppayil S.M. (2014). A status review on the medicinal properties of essential oils. Ind. Crops Prod..

[17] Trifan A., Luca S.V., Greige-Gerges H., Miron A., Gille E., Aprotosoaie A.C. (2020). Recent advances in tackling microbial multidrug resistance with essential oils: Combinatorial and nano-based strategies. Crit. Rev. Microbiol..

[18] Ribeiro-Santos R., Andrade M., Sanches-Silva A., de Melo N.R. (2018). Essential oils for food application: Natural substances with established biological activities. Food Bioprocess. Technol..

[19] Cutillas A.B., Carrasco A., Martinez-Gutierrez R., Tomas V., Tudela J. (2018). Thyme essential oils from Spain: Aromatic profile ascertained by GC–MS, and their antioxidant, anti-lipoxygenase and antimicrobial activities. J. Food Drug Anal..

[20] Gonçalves M.J., Cruz M.T., Cavaleiro C., Lopes M.C., Salgueiro L. (2010). Chemical, antifungal and cytotoxic evaluation of the essential oil of *Thymus zygis* subsp. sylvestris. Ind. Crops Prod..

[21] Lagha R., Abdallah F.B., AL-Sarhan B.O., Al-Sodany Y. (2019). Antibacterial and biofilm inhibitory activity of medicinal plant essential oils against *Escherichia coli* isolated from UTI patients. Molecules.

[22] Debonne E., Vermeulen A., Van Bockstaele F., Soljic I., Eeckhout M., Devlieghere F. (2019). Growth/no-growth models of in-vitro growth of *Penicillium paneum* as a function of thyme essential oil, pH, aw, temperature. Food Microbiol..

[23] Yang V.W., Clausen C.A. (2007). Antifungal effect of essential oils on southern yellow pine. Int. Biodeterior. Biodegrad..

[24] Santoyo S., Jaime L., García-Risco M.R., Lopez-Hazas M., Reglero G. (2014). Supercritical fluid extraction as an alternative process to obtain antiviral agents from thyme species. Ind. Crops Prod..

[25] Machado M., Dinis A.M., Salgueiro L., Cavaleiro C., Custódio J.B.A., Do Céu Sousa M. (2010). Anti-*Giardia* activity of phenolic-rich essential oils: Effects of *Thymbra capitata*, *Origanum virens*, *Thymus zygis* subsp. sylvestris, and Lippia graveolens on trophozoites growth, viability, adherence, and ultrastructure. Parasitol. Res..

[26] Sangha J.S., Astatkie T., Cutler G.C. (2017). Ovicidal, larvicidal, and behavioural effects of some plant essential oils on diamondback moth (Lepidoptera: Plutellidae). Can. Entomol..

[27] Park C.G., Jang M., Yoon K.A., Kim J. (2016). Insecticidal and acetylcholinesterase inhibitory activities of Lamiaceae plant essential oils and their major components against *Drosophila suzukii* (Diptera: Drosophilidae). Ind. Crops Prod..

[28] Scherer R., Godoy H.T. (2009). Antioxidant activity index (AAI) by the 2,2-diphenyl-1-picrylhydrazyl method. Food Chem..

[29] Roudashti S., Zeighami H., Mirshahabi H., Bahari S., Soltani A., Haghi F. (2017). Synergistic activity of sub-inhibitory concentrations of curcumin with ceftazidime and ciprofloxacin against *Pseudomonas aeruginosa* quorum sensing related genes and virulence traits. World J. Microbiol. Biotechnol..

[30] Algburi A., Comito N., Kashtanov D., Dicks L.M.T., Chikindas M.L. (2017). Control of biofilm formation: Antibiotics and beyond. Appl. Environ. Microbiol..

[31] Perricone M., Arace E., Corbo M.R., Sinigaglia M., Bevilacqua A. (2015). Bioactivity of essential oils: A review on their interaction with food components. Front. Microbiol..

[32] Idrees M., Sawant S., Karodia N., Rahman A. (2021). *Staphylococcus aureus* biofilm: Morphology, genetics, pathogenesis and treatment strategies *Int*. J. Environ. Res. Public Health.

[33] Vieira M., Bessa L.J., Martins M.R., Arantes S., Teixeira A.P.S., Mendes Â., Da Costa P.M., Belo A.D.F. (2017). Chemical composition, antibacterial, antibiofilm and synergistic properties of essential oils from *Eucalyptus globulus* LABILL. and seven Mediterranean aromatic plants. Chem. Biodivers..

[34] Dorman H.J.D., Deans S.G. (2004). Chemical composition, antimicrobial and *in vitro* antioxidant properties of *Monarda citriodora* var. *citriodora*, *Myristica fragrans*, *Origanum vulgare* ssp. *hirtum*, *Pelargonium* sp. and *Thymus zygis* oils.. J. Essent. Oil Res..

[35] Pina-Vaz C., Rodrigues A.G., Pinto E., Costa-de-Oliveira S., Tavares C., Salgueiro L., Cavaleiro C., Gonçalves M., Martinez-de-Oliveira J. (2004). Antifungal activity of *Thymus* oils and their major compounds. J. Eur. Acad. Dermatol. Venereol..

[36] Ballester-Costa C., Sendra E., Fernández-López J., Pérez-Álvarez J.A., Viuda-Martos M. (2013). Chemical composition and in vitro antibacterial properties of essential oils of four *Thymus* species from organic growth. Ind. Crops Prod..

[37] Sánchez-Hidalgo M., Montalbán-López M., Cebrián R., Valdivia E., Martínez-Bueno M., Maqueda M. (2011). AS-48 bacteriocin: Close to perfection. Cell. Mol. Life Sci..

[38] Rota C., Herrera A., Martınez R.M., Sotomayor J.A., Jordán M.J. (2008). Antimicrobial activity and chemical composition of *Thymus vulgaris*, *Thymus zygis* and *Thymus hyemalis* essential oils. Food Control.

[39] Andrés M.F., González-coloma A., Muñoz R., De la Peña F., Julio L.F., Burillo J. (2018). Nematicidal potential of hydrolates from the semi industrial vapor-pressure extraction of Spanish aromatic plants. Environ. Sci. Pollut. Res..

[40] Marinković J., Ćulafić D.M., Nikolić B., Đukanović S., Marković T., Tasić G., Ćirić A., Marković D. (2020). Antimicrobial potential of irrigants based on essential oils of *Cymbopogon martinii* and *Thymus zygis* towards in vitro multispecies biofilm cultured in *ex vivo* root canals. Arch. Oral Biol..

[41] Solarte A.L., Astorga R.J., De Aguiar F.C., De Frutos C., Barrero-Domínguez B., Huerta B. (2018). Susceptibility Ddistribution to essential oils of *Salmonella enterica* strains involved in animal and public health and comparison of the Typhimurium and Enteritidis Serotypes. J. Med. Food.

[42] Youdim K.A., Deans S.G., Finlayson H.J. (2002). The antioxidant properties of thyme (*Thymus zygis* L.) essential oil: An inhibitor of lipid peroxidation and a free radical scavenger. J. Essent. Oil Res..

[43] Carrasco A., Tomas V., Tudela J., Miguel M.G. (2015). Comparative study of GC-MS characterization, antioxidant activity and hyaluronidase inhibition of different species of *Lavandula* and *Thymus* essential oils. Flavour Fragr. J..

[44] Jordán M.J., Martínez R.M., Martínez C., Moñino I., Sotomayor J.A. (2009). Polyphenolic extract and essential oil quality of *Thymus zygis* ssp. *gracilis* shrubs cultivated under different watering levels. Ind. Crops Prod..

[45] Ballester-Costa C., Sendra E., Fernández-López J., Pérez-Álvarez J.A., Viuda-Martos M. (2017). Assessment of antioxidant and antibacterial properties on meat homogenates of essential oils obtained from four *Thymus* species achieved from organic growth. Foods.

[46] Escobar A., Pérez M., Romanelli G., Blustein G. (2020). Thymol bioactivity: A review focusing on practical applications. Arab. J. Chem..

[47] Ghabraie M., Vu K.D., Tata L., Salmieri S., Lacroix M. (2016). Antimicrobial effect of essential oils in combinations against five bacteria and their effect on sensorial quality of ground meat. LWT Food Sci. Technol..

[48] Abdallah F.B., Lagha R., Gaber A. (2020). Biofilm inhibition and eradication properties of medicinal plant essential oils against methicillin-resistant *Staphylococcus aureus* clinical isolates. Pharmaceuticals.

[49] Brochot A., Guilbot A., Haddioui L., Roques C. (2017). Antibacterial, antifungal, and antiviral effects of three essential oil blends. Microbiol. Open.

[50] Wang X., Shen Y., Thakur K., Han J., Zhang J.G., Hu F., Wei Z.J. (2020). Antibacterial activity and mechanism of ginger essential oil against *Escherichia coli* and *Staphylococcus aureus*. Molecules.

[51] Bilia A.R., Guccione C., Isacchi B., Righeschi C., Firenzuoli F., Bergonzi M.C. (2014). Essential oils loaded in nanosystems: A developing strategy for a successful therapeutic approach. Evid. Complement. Altern. Med..

[52] Clinical and Laboratory Standards Institute (CLSI) (2021). Performance Standards for Antimicrobial Susceptibility Testing.

[53] Palaniappan K., Holley R.A. (2010). Use of natural antimicrobials to increase antibiotic susceptibility of drug resistant bacteria. Int. J. Food Microbiol..

[54] Wang L.H., Zhang Z.H., Zeng X.A., Gong D.M., Wang M.S. (2017). Combination of microbiological, spectroscopic and molecular docking techniques to study the antibacterial mechanism of thymol against Staphylococcus aureus: Membrane damage and genomic DNA binding. Anal. Bioanal. Chem..

[55] Shi C., Zhao X., Yan H., Meng R., Zhang Y., Li W., Liu Z., Guo N. (2016). Effect of tea tree oil on *Staphylococcus aureus* growth and enterotoxin production. Food Control.

[56] Qiu J., Li H., Su H., Dong J., Luo M., Wang J., Leng B., Deng Y., Liu J., Deng X. (2012). Chemical composition of fennel essential oil and its impact on *Staphylococcus aureus* exotoxin production. World J. Microbiol. Biotechnol..

[57] Coimbra A.T., Luís Â.F.S., Batista M.T., Ferreira S.M.P., Duarte A.P.C. (2020). Phytochemical characterization, bioactivities evaluation and synergistic effect of *Arbutus unedo* and *Crataegus monogyna* extracts with amphotericin B. Curr. Microbiol..

[58] Luís Â., Duarte A.P., Pereira L., Domingues F. (2017). Chemical profiling and evaluation of antioxidant and anti-microbial properties of selected commercial essential oils: A comparative study. Medicines.

[59] Duarte A., Luís Â., Oleastro M., Domingues F.C. (2016). Antioxidant properties of coriander essential oil and linalool and their potential to control *Campylobacter* spp. Food Control.

[60] Ferreira S., Domingues F. (2016). The antimicrobial action of resveratrol against *Listeria monocytogenes* in food-based models and its antibiofilm properties. J. Sci. Food Agric..

[61] Stepanović S., Ćirković I., Ranin L., Švabić-Vlahović M. (2004). Biofilm formation by *Salmonella* spp. and *Listeria monocytogenes* on plastic surface. Lett. Appl. Microbiol..

[62] Duarte A., Alves A.C., Ferreira S., Silva F., Domingues F.C. (2015). Resveratrol inclusion complexes: Antibacterial and anti-biofilm activity against *Campylobacter* spp. and *Arcobacter butzleri*. Food Res. Int..

[63] Asensio C.M., Quiroga P.R., Al-Gburi A., Huang Q., Grosso N.R. (2020). Rheological behavior, antimicrobial and Qquorum sensig inhibition study of an argentinean oregano essential oil nanoemulsion. Front. Nutr..

[64] Luís Â., Silva F., Sousa S., Duarte A.P., Domingues F. (2014). Antistaphylococcal and biofilm inhibitory activities of gallic, caffeic, and chlorogenic acids. Biofouling.

[65] Silva F., Ferreira S., Duarte A., Mendona D.I., Domingues F.C. (2011). Antifungal activity of *Coriandrum sativum* essential oil, its mode of action against *Candida* species and potential synergism with amphotericin B. Phytomedicine.

